# RNA-Guided Genome Editing in *Drosophila* with the Purified Cas9 Protein

**DOI:** 10.1534/g3.114.012179

**Published:** 2014-07-01

**Authors:** Jeong-Soo Lee, Su-Jin Kwak, Jungeun Kim, Ae-Kyeong Kim, Hae Min Noh, Jin-Soo Kim, Kweon Yu

**Affiliations:** * Neurophysiology Research Group, Bionano Center, Korea Research Institute of Bioscience and Biotechnology (KRIBB), Daejeon 305-806, Korea; † Functional Genomics Department, Korea University of Science and Technology (KUST), Daejeon 305-333, Korea; ‡ National Creative Research Initiatives Center for Genome Engineering and Department of Chemistry, Seoul National University, Seoul 151-747, Korea; § Center for Genome Engineering, Institute for Basic Science, Daejeon, Korea

**Keywords:** Cas9 protein, RGEN, *Drosophila*

## Abstract

We report a method for generating *Drosophila* germline mutants effectively via injection of the complex of the purified Cas9 protein, tracrRNA, and gene-specific crRNAs, which may reduce delayed mutations because of the transient activity of the Cas9 protein, combined with the simple mutation detection in GO founders by the T7E1 assay.

In the past few years, new technologies to knockout the genes of interests have been developed, including zinc-finger nucleases (ZFNs) (Kim *et al.* 2011), transcription activator-Like effector nucleases (TALENs) (Kim *et al.* 2013), and RNA-guided engineered nucleases (RGENs) derived from the clustered regularly interspaced short palindromic repeat RNA/CRISPR-associated (CRISPR/Cas) system (Gaj *et al.* 2013). Among them, RGENs are the latest gene knockout tools that were originally identified as an acquired immunity-like system in bacteria to protect the host from invading viruses or plasmids (Wiedenheft *et al.* 2012; Mali *et al.* 2013).

RGENs consist of three components: Cas9 endonuclease, CRISPR RNA (crRNA) that complementarily binds to the target site of the genomic DNA, and *trans*-activating CRISPR RNA (tracrRNA). This Cas9/tracrRNA/crRNA tripartite complex or its modified version, the bipartite complex consisting of Cas9/chimeric RNA (also called single-guided RNA or sgRNA), can cleave chromosomal DNA in a targeted manner and induce mutations efficiently in the vicinity of target sites in model organisms such as mouse, zebrafish, and *C. elegans* or in cell lines via the error-prone nonhomologous end-joining (NHEJ) DNA repair system (Cho *et al.* 2013a,b; Mali *et al.* 2013; Wang *et al.* 2013; Sung *et al.* 2014). RGENs were also successfully applied to *Drosophila melanogaster*, another major genetic model organism, but with differences in detailed methodologies; Cas9 was delivered as DNA plasmid (Gratz *et al.* 2013), mRNA (Bassett *et al.* 2013; Yu *et al.* 2013), or transgenic expression under a germ-cell–specific promoter (Kondo and Ueda 2013; Ren *et al.* 2013). Similarly, sgRNA was injected as DNA (Gratz *et al.* 2013a; Ren *et al.* 2013), RNA (Bassett *et al.* 2013; Yu *et al.* 2013), and transgenic expression under a universal promoter (Kondo and Ueda 2013). These approaches showed various efficiencies in terms of germ-line transmission rates and the percentages of mutants in F1 embryos (Gratz *et al.* 2013b; Bassett and Liu 2014; Beumer and Carroll 2014).

Here, we used an alternative approach in which the *in vitro* pre-formed ribonucleoprotein complex of the purified Cas9 protein, *in vitro* transcribed tracrRNA, and target gene–specific crRNAs was injected into the *Drosophila* embryos to knockout the gene of interest and validated the mutation-inducing efficiency based on the T7 endonuclease I (T7E1) assay in G0 founders and F1 mutants. The mutational sequences were later confirmed by direct sequencing. Our results indicate that our CRISPR/tracrRNA/crRNA complex injection induced loss-of-function mutations efficiently with low toxicity. The combined usage of the purified Cas9 protein and the T7E1 assay can help to validate candidate crRNAs for injection to choose efficient crRNAs *in vitro* and *in vivo*. In addition, the pre-formed ribonucleoprotein complex consisting of the purified Cas9 protein/tracrRNA/crRNA shown to act immediately after injection and degrade rapidly may alleviate delayed mutation events, helping reduce off-target effects and mosaicism by RGENs.

## Results and Discussion

**Figure 1:**
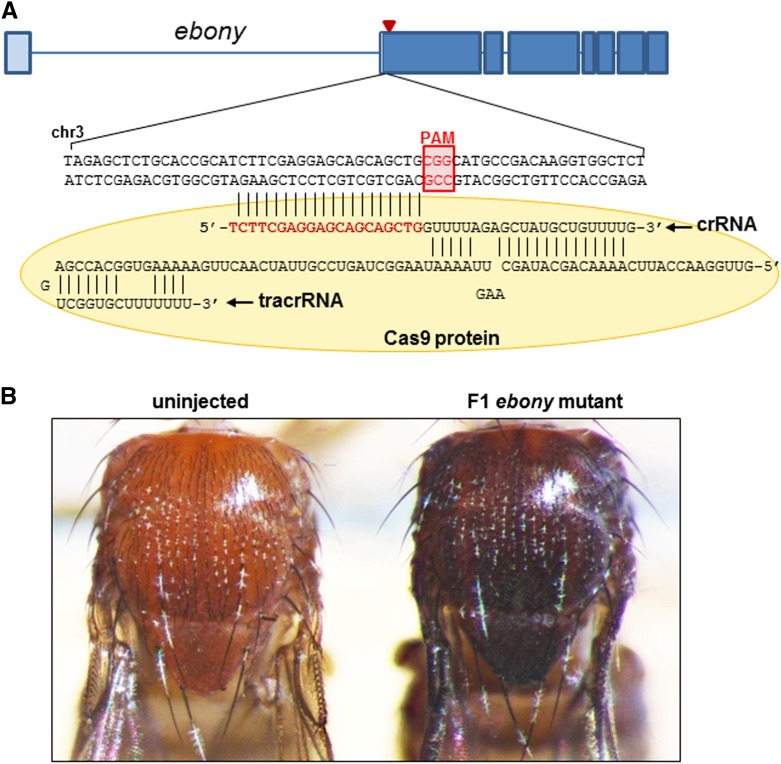
Generating novel *ebony* mutants using Cas9 protein/TracrRNA/crRNA complex. (A) Genomic structure of the *ebony* gene. The target nucleotide sequence upstream of the start codon (red triangle) neighboring the PAM sequence is complementary to the 5′ end of 20 bp of crRNA (shown in red). TracrRNA and Cas9 protein (shown as yellow circle) form the ribonucleoprotein complex along with gene-specific crRNA. (B) Compared to the brown body color of the wild-type adults (uninjected; left), the *ebony* F1 mutant (right) shows dark black body color, the phenotype due to the disrupted *ebony* gene by the injection of Cas9 protein/TracrRNA/*ebony*–specific crRNA complex.

To test whether the tripartite ribonucleoprotein (RNP) complex of purified Cas9 protein, tracrRNA, and gene-specific crRNA (“RGEN-RNP” below) can generate mutations in the target sites of *Drosophila* genome, we first chose the *ebony* (*e*) gene in the third chromosome dictating the body color (Figure 1B). We designed two *e*-specific crRNAs that can bind to the second exon upstream of the start codon (Figure 1A). Syncytial blastoderm-stage *w^1118^* embryos were microinjected with each complex of Cas9/RNAs at different concentrations, grown to adulthood, and individually crossed to *D*/*e*, *Ser*, *TM3* to see if mutations were generated and transmitted through the germline cells into the F1 generation (see Supporting Information, Figure S1A for the cross scheme). It was found that the complex with only one of the two tested crRNAs gave rise to the F1 progeny with the *e* mutant phenotype (Figure 1B and Table 1). With the injection of crRNA(330 ng)-containing RGEN-RNP, 26 “G0 founder” flies, defined as adult flies that yielded at least one F1 mutant, out of 191 flies screened produced the F1 mutants (germline transmission rate, defined as the percentage of G0 founder/G0 adults screened, 26/191 = 13.61%) (Table 1), indicating that this method is capable of inducing loss-of-function mutations efficiently at target sites. Such rate decreased significantly at a higher concentration of crRNA (660 ng crRNA-containing complex, 2/77 = 2.6%) (Table 1), suggesting that injected doses of the RGEN-RNP are critical for the efficient induction of mutations. The percentages of the F1 mutants in individual F1 offspring clutches from G0 founders varied from 1.8% to 25% with the 330 ng crRNA-containing RGEN-RNP (Table 1).

**Table 1:** Efficiency of germline-transmitted mutations

crRNA (ng)	Number of Injected Embryos	Number of G0 Adults Screened		Number of Mosaic G0 Adults	Number of G0 Founder	Germline Transmission Rate (%)	Percentage of Mutants in F1 Offspring (%)
		Female	Male				
*e (660)*	400	42	35	0	2	2.6% (2/77)	1.9–3.5
*e (330)*	900	110	81	0	26	13.61% (26/191)	1.8–25
*sn (660)*	400	64	53	3	5	7.81% (5/64)	1.7–3.5
*sn (330)*	400	20	16	3	4	20% (4/20)	2.4–8.4
*sn (130)*	600	82	74	1	0	0	0

G0 founders yielded at least one F1 mutant, the germline transmission rate is defined as the percentage of G0 founders/G0 adults screened, and the percentage of mutants in F1 offspring means the percentage of the F1 mutant/F1 adults in each clutch.

**Figure 2:**
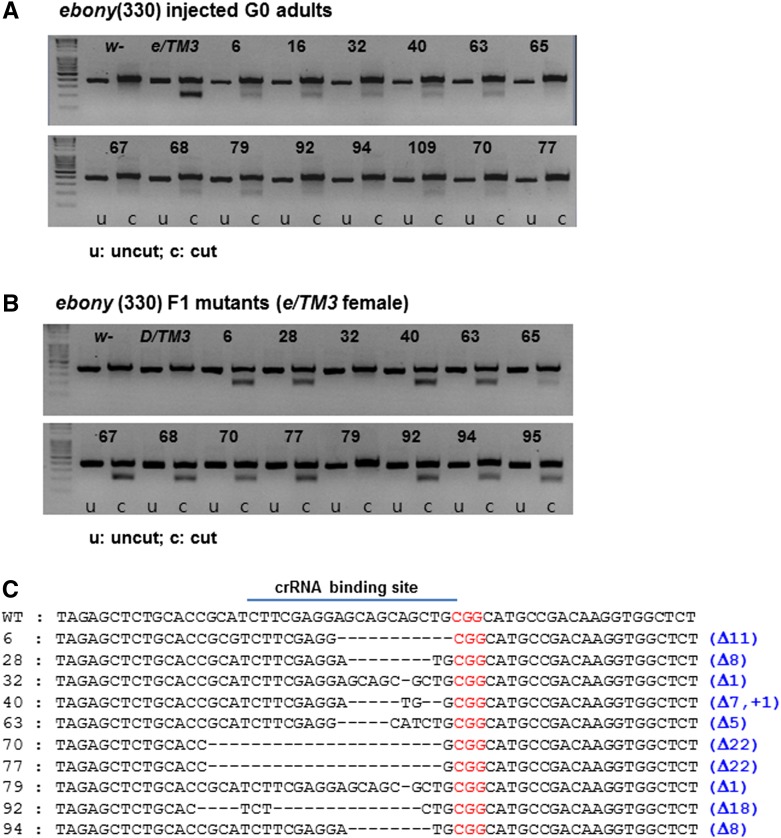
Identification of *ebony* mutations by T7E1 assay and direct sequencing. (A) DNA-mismatched mutations induced by the injection of Cas9/RNA complex containing 330 ng of *e* crRNA were detected in G0 adult flies by the T7E1 assay. Each sample consists of two lanes as a pair in the agarose gel: the left lane is “uncut (u),” meaning no treatment, whereas the right is “cut (c),” digested by the T7E1 endonuclease. The numbers on top of each sample represent independently injected individual adult flies. The “cut” lanes show additional lower DNA bands in addition to ∼400 bp of uncut PCR-amplified DNA because mismatched DNA induced by the Cas9/RNA complex were digested by the T7E1. Diverse intensities of lower bands presumably reflect different degrees of mosaic mutations. *w−* is the negative control for the T7E1 assay, whereas *e/TM3*, heterozygous for *ebony*, is the positive control. (B) The T7E1 assay of individual F1 mutant flies. The format of the gel is similar to (A). The same numbers of samples in (A) and (B) indicate the relationship of parents and their offspring. Both *w−* and *D/TM3* are negative controls. (C) Direct sequencing of the target site in individual F1 mutants revealed various deletion mutations. The numbers on the left indicate the individuals in (B) from which the DNA sequences were derived. Although rare, the deletion and insertion occurred simultaneously during nonhomologous end-joining DNA repair process (in the case of number 40). CGG in red denotes the PAM sequence.

To detect mutations and evaluate the efficiency induced by the RGEN-RNP on injection, we used the T7E1 assay that can recognize and cleave mismatched base pairs of wild-type and mutation-harboring DNA (Kim *et al.* 2009; see supplementary *Materials and Methods* in File S1 for details). Injection of the RGEN-RNP with 330 ng of the *e*-specific crRNA produced cleaved bands with various intensities in individual G0 founders by the T7E1 assay (Figure 2A). Such bands are thought to reflect different amounts of mismatched DNAs mostly due to the somatic mutations in injected individuals. Germline-transmitted F1 mutants were also confirmed by similar cleaved patterns but with more distinct bands in the T7E1 assay, presumably because they are heterozygous for mutations (Figure 2B). These results show that the simple and straightforward T7E1 assay can be used to detect mutations induced by RGEN-RNPs in the G0 and F1 generations of *Drosophila*. This assay can be also useful to decide on the best-working crRNA among multiple candidate crRNAs when tested in G0 mosaic individuals.

To identify the nature of mutations induced by RGEN-RNP injection, we subcloned and sequenced the target genomic site of the *ebony* gene in the F1 mutant individuals previously confirmed by the T7E1 assay. A series of small deletion mutations, ranging from 1 base pair (bp) to 22 bp, was identified. Most of them cause the early stop of translation because of out-of-frame mutations and are expected to generate a nonfunctional protein (Figure 2C).

**Figure 3:**
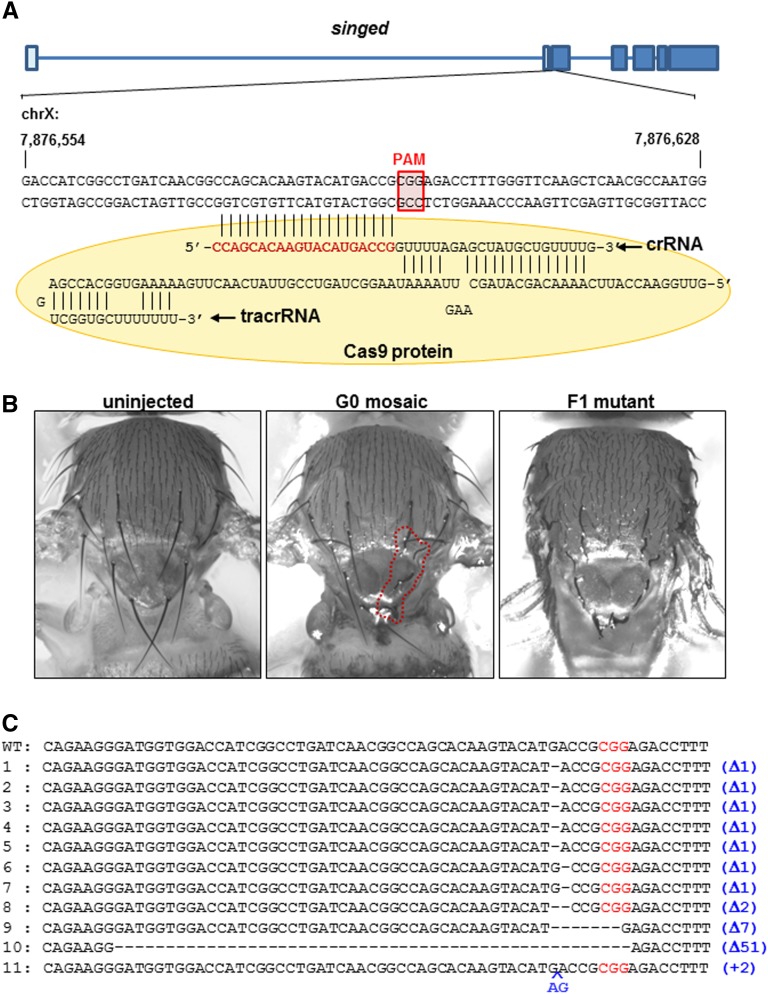
Identification of novel *singed* mutations. (A) Genomic structure of the *singed* gene. Similar to the *ebony* gene in Figure 1A, the target nucleotide sequence neighboring the PAM sequence is complementary to the 5′ end of 20 bp of crRNA (shown in red). TracrRNA and Cas9 protein (shown in yellow circle) *singed*-specific crRNA form the ribonucleoprotein complex. (B) Compared to the straight bristles of the wild-type (uninjected; left) the *singed* F1 mutants show the gnarled bristles (right), the phenotype due to the disruption of the *singed* gene by the injection of Cas9 protein/TracrRNA/*singed*–specific crRNA complex. Interestingly, this injection leads to gnarled bristles in some G0 founders in a mosaic fashion (middle, red circle), indicating somatic mutations induced by the Cas9/RNA complex. (C) Direct sequencing of the target site in individual F1 mutants reveals various insertion/deletion mutations. The numbers on the left side indicate individual F1 mutants. Deletions were the prevailing mutations while insertion was identified in one individual (blue in number 11). CGG in red denotes the PAM sequence.

We further confirmed our RGEN-RNP–mediated gene knockout method for another gene, *singed* (*sn*), which is an X-chromosome gene that determines bristle formation (Figure 3, A and B). Injection of RGEN-RNP produced partially gnarled bristles even in the G0 adults as mosaic patterns, suggesting the induction of somatic mutations by *sn*-specific RGEN-RNP (Figure 2B and Table 1). Interestingly, one of the three mosaic G0 adult flies was the female fly, indicating the biallelic mutations in X chromosomes, suggesting high activity of the injected RGEN-RNP. When injected G0 adult female flies were individually crossed with wild-type males (Figure S1B) and the gnarled bristle phenotype was examined in F1 males, it was found that 20% (4/20, 330-ng crRNA injection) and 7.81% (5/64, 660-ng crRNA injection) of the G0 adult females carried the loss-of-function mutations in the *sn* gene of germline cells (Table 1). Direct sequencing encompassing the *sn* target site of F1 males confirmed insertion/deletion (indel) mutations ranging from 51 bp deletion to 2 bp insertion (Figure 3C). Some of these mutations were also confirmed by the T7E1 assay, showing the cleaved patterns (Figure S2).

Taken together, our results demonstrated that not only the RGEN-RNP-mediated (consisting of purified Cas9 protein, tracrRNA, and gene-specific crRNA) gene knockout method efficiently induces loss-of-function mutations for the *Drosophila* genes but also an optimal dose of the complex is required for its maximal activity because their knockdown efficiency appears to be dose-dependent (Table 1).

In using the CRISPR/Cas9 system to generate *Drosophila* knockouts, our study adopted two methodologies different from previous approaches: we used the purified Cas9 protein instead of *cas9* DNA plasmid (Gratz *et al.* 2013a), *in vitro* transcribed *cas9* RNA (Bassett *et al.* 2013; Yu *et al.* 2013), or germline-specific *nos-cas9* transgenic fly (Kondo and Ueda 2013; Ren *et al.* 2013), and we the combined tracrRNA and gene-specific crRNA, which were prepared separately instead of using single-guided (or chimeric) RNA (sgRNA) (Jinek *et al.* 2012).

The ribonucleoprotein complex consisting of purified Cas9 protein and sgRNA was successfully applied for generating gene knockouts in *C. elegans* (Cho *et al.* 2013) and recently in mice and zebrafish (Sung *et al.* 2014), but it has not been tested in *Drosophila* thus far. The germline transmission rate (the percentage of G0 founders that yields at least a single F1 mutant) in our study (ranging from 2.6% to 20%) is relatively lower than that reported in other species using Cas9 protein/sgRNA complex (more than 20%) (Sung *et al.* 2014) and other *Drosophila* studies using TALEN RNA for *trh* gene (17% to 39%) (Kondo *et al.* 2014), cas9 RNA/sgRNA injection for *yellow* or *w* gene (12.4% to 68%) (Bassett *et al.* 2013, Yu *et al.* 2013), and germline-specific *nos-cas9* transgenic expression for *w*, neuropeptides, and miRNAs (88.2% to 100%) (Ren *et al.* 2013; Kondo and Ueda 2013). In contrast, our germline transmission rate is higher than that using plasmids for *cas9* and sgRNA (∼5%) (Gratz *et al.* 2013a). The difference in these rates may originate from the different efficiency of sgRNA *vs*. tracrRNA/crRNA or the distinctive nature of the target genes tested in different studies (*e.g.*, some genes are more targetable than others). The inherent difference of *Drosophila* from other species in genome editing processes may also contribute to such differences. Of note, it has been reported that the tracrRNA/crRNA combination similar to the one used in our study is at least as efficient as sgRNA and it may even contribute to the reduction of off-target effects (Cho *et al.* 2014), which is one of the major concerns of the Cas9/RNA system.

Despite the relatively low efficiency of the RGEN-RNP injection in our study compared to the germline-specific *nos-cas9* transgenic system or *cas9* RNA injection, using the purified Cas9 protein pre-formed with tracrRNA/crRNA (or sgRNA) instead of *cas9* DNA or RNA has a few advantages over other methods. First, the transient activity of the RGEN-RNP system can help alleviate delayed mutation events on delivery. Continuous expression of *cas9* DNA in germ cells (Kondo and Ueda 2013; Ren *et al.* 2013) or its fairly stable RNA injection (Bassett *et al.* 2013; Sung *et al.* 2014; Yu *et al.* 2013) into developing embryos may elicit mutational events for a prolonged period of the development. This can increase the chance of unwanted off-target effects as well as on-target mutations and mutational mosaicism in independent cells of embryos, leading to multiple mutant alleles in following generations and potentially complicating further analyses. Contrary to the transgenic expression and RNA injection of *cas9*, the action of the purified Cas9 protein is expected to be rapid and transient because of its delivery as a form of protein and its short half-life. We have recently shown in human cell lines that the RGEN-RNP system acts immediately on delivery and the purified Cas9 protein was degraded within 24 hr after being applied to cultured cells (S. Kim *et al.* 2014). In this report, we also validated that the RGEN-RNP system induced off-target mutations quite rarely (up to 13-fold less than Cas9 plasmid did; confirmed by deep sequencing) without compromising on-target mutation efficiency (S. Kim *et al.* 2014). However, direct comparisons of mutational mosaicism in animal models by the RGEN-RNP and other forms of cas9 remain to be determined.

Another advantage of using the RGEN-RNP system is that designed sgRNAs or crRNAs can be validated for their efficiency for inducing mutations to select the best ones by using the purified Cas9 protein in an *in vitro* cleavage assay before the actual transfection/injection into cells or embryos is embarked (Sung *et al.* 2014). Furthermore, such a system can be exploited to genotype the heterozygous and homozygous mutant progenies as an alternative RFLP (restriction fragment length polymorphism) without requiring specific restriction enzymes and recognized sites (J. M. Kim *et al.* 2014). However, our RGENP-RNP method requires an additional step of preparing recombinant Cas9 protein using a bacterial *in vitro* translation system (S. Kim *et al.* 2014), and the relative instability of Cas9 protein compared to DNA or RNA of Cas9 for long-term storage may be practically problematic.

The selection of target gene–specific crRNAs or sgRNAs that work most efficiently *in vivo* among multiple candidates predicted from bioinformatics is one of the key requirements to generate mutants of animal models using the CRISPR/Cas9 system. Several methods have been developed that allow the detection of mutations in the target DNA induced by genome editing tools *in vivo*, preferably in the G0 founders. For example, Bassett *et al.* (2013) and Gratz *et al.* (2013a) adopted high-resolution melting analysis (HRMA) and the surveyor kit (Transgenomic, Inc), respectively. The T7E1 assay used in this study is similar to the surveyor kit in that DNA mismatches are detected by digestion of a specialized endonuclease, but it is simpler and can detect mutations using genomic DNA from *Drosophila* G0 founders (Figure 2).

Our current study proposes a strategy to generate *Drosophila* mutants for a certain gene. First, an investigator designs multiple crRNAs (or sgRNAs) that are predicted to show minimal off-target effects using web-based software such as http://flyrnai.org/crispr/ (Hsu *et al.* 2013) and http://crispr.mit.edu/ (Ren *et al.* 2013). The potential off-target sites can be checked at this step (http://www.rgenome.net/cas-offinder/) (Bae *et al.* 2014). Simultaneously, the purified Cas9 protein is prepared by *in vitro* translation. Second, the efficiency of multiple crRNAs (or sgRNA) with different doses and ratios with Cas9 protein are verified and the best-working ones are selected by performing the aforementioned *in vitro* cleavage assay using a RGEN-RNP (mentioned above) and the T7E1 assay after PCR using genomic DNA from G0 individuals as the DNA template. If one prefers the *in vivo* assay and wants to save time, then the *in vitro* cleavage assay can be skipped. Next, G0 founders exhibiting cleaved patterns by the T7E1 assay are crossed to identify lines that carry germline-transmitted mutations. Finally, heterozygous and homozygous mutant progenies in the following generations can be genotyped using either RGEN-RNP RFLP or T7E1 assays, and the mutagenesis efficiency can be calculated.

In summary, our method reported in this study is a valid alternative to be considered for generating *Drosophila* mutants with a reasonable efficiency and as little time and effort as possible, especially when the prevention of delayed mutation events is preferred to reduce the off-target effects and mosaicism.

## Supplementary Material

Supporting Information
