# Supplementary material for: RNA-Guided Genome Editing in Drosophila with the Purified Cas9 Protein
Source: G3 (Bethesda). 2014 Jul 1;4(7):1291–5. doi: 10.1534/g3.114.012179 (PMC4455777; doi:10.1534/g3.114.012179)
Supplement: Supporting Information [file supp_4_7_1291__index.html]

RNA-Guided Genome Editing in Drosophila with the Purified Cas9 Protein — Supporting Information 

# RNA-Guided Genome Editing in *Drosophila* with the Purified Cas9 Protein

## Supporting Information for Lee *et al.*, 2014

**Files in this Data Supplement:**

- Supporting Information - Figures S1-S2 and File S1 (PDF, 175 KB)
- Figure S1 - Genetic schemes for mutant screening. (PDF, 48 KB)
- Figure S2 - Validation of *sn* mutants by the T7E1 assay. (PDF, 112 KB)
- File S1 - Supplementary Materials and Methods (PDF, 37 KB)
