# Supplementary material for: RNA-Guided Genome Editing in Drosophila with the Purified Cas9 Protein
Source: G3 (Bethesda). 2014 Jul 1;4(7):1291–5. doi: 10.1534/g3.114.012179 (PMC4455777; doi:10.1534/g3.114.012179)
Supplement: Supporting Information [file supp_g3.114.012179_FigureS1.pdf]

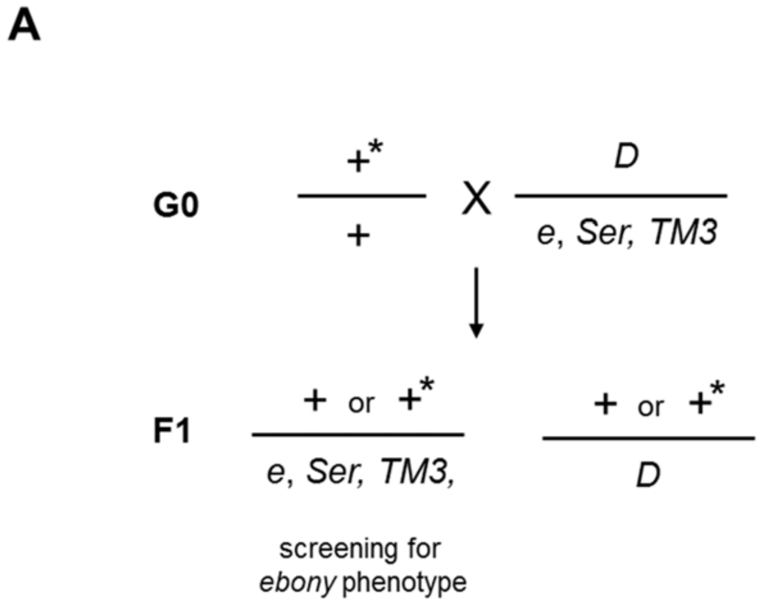

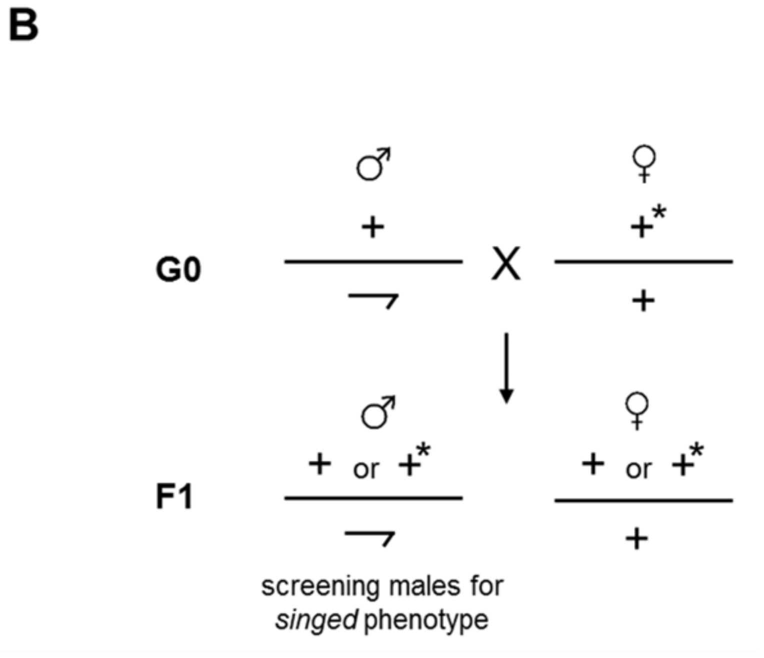

Figure S1 Genetic schemes for mutant screening. (A) A screening scheme for screening *ebony* mutants. In order to detect the mutant phenotype of *ebony*, an autosomal gene, in the F1 generation, Cas9/RNA complex-injected G0 flies were crossed with *ebony* heterozygous adults with double-balanced chromosome *D / e, Ser, TM3*. Since *ebony* is linked to the *Ser, TM3* balancer, only adults showing the serrated wing phenotype (also heterozygous for *ebony*) were monitored in the F1

generation to see if they exhibited the *ebony* phenotype. This indicates that the *ebony* gene in the homologous chromosome descended from injected G0 adults carried the mutation induced by the injection of Cas9/RNA complex. (B) Screening scheme for *singed* mutants. In order to detect the mutant phenotype of *singed,* an X chromosome-linked gene, in the F1 generation, Cas9/RNA complex-injected G0 female flies were crossed with wild-type flies and F1 males were screened for the *singed* phenotype.
