# Supplementary material for: RNA-Guided Genome Editing in Drosophila with the Purified Cas9 Protein
Source: G3 (Bethesda). 2014 Jul 1;4(7):1291–5. doi: 10.1534/g3.114.012179 (PMC4455777; doi:10.1534/g3.114.012179)
Supplement: Supporting Information [file supp_g3.114.012179_FigureS2.pdf]

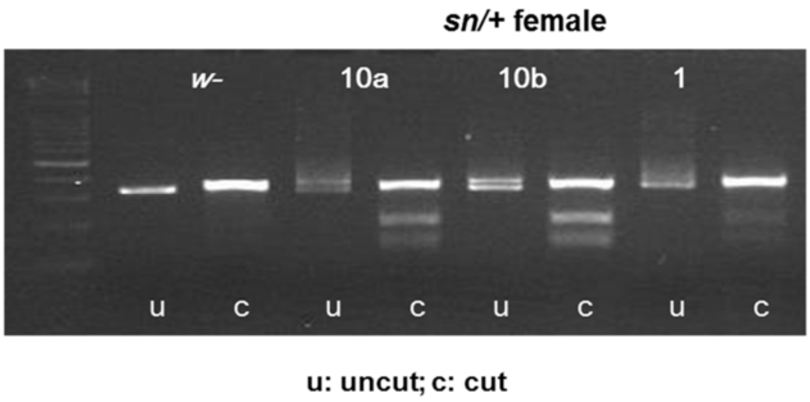


```
WT: CAGAAGGGATGGTGGACCATCGGCCTGATCAACGGCCAGCACAAGTACATGACCGCGGAGACCTTT
1 : CAGAAGGGATGGTGGACCATCGGCCTGATCAACGGCCAGCACAAGTACAT-ACCGCGGAGACCTTT (Δ1)
10a:CAGAAGG---------------------------------------------------AGACCTTT (Δ51)
10b:CAGAAGG---------------------------------------------------AGACCTTT (Δ51)
```

Figure S2 Validation of *sn* mutants by the T7E1 assay. Each sample consists of two lanes as a pair, ‘uncut’ and ‘cut’ lanes for the T7E1 assay similar to Fig. 2A and 2B. The sample numbers in the gel (top) and in the left side of DNA sequences (bottom) of mutant progeny in this figure matched those of F1 mutants in Fig. 3C (numbers 1 and 10). Sample numbers 10a and 10b indicate two independent mutant progeny flies for the same F1 parent number 10. Compared to uncut lanes (“u”), mutant progeny exhibited cleaved patterns (“c”) by the T7E1 digestion indicating the presence of DNA mismatches due to mutations. *w-* is the negative control.
