# Supplementary material for: RNA-Guided Genome Editing in Drosophila with the Purified Cas9 Protein
Source: G3 (Bethesda). 2014 Jul 1;4(7):1291–5. doi: 10.1534/g3.114.012179 (PMC4455777; doi:10.1534/g3.114.012179)
Supplement: Supporting Information [file supp_g3.114.012179_FileS1.pdf]

File S1

Supplementary Materials and Methods

Preparation of Cas9 protein and tracrRNA /gene-specific crRNA

The Cas9 protein and tracrRNA/gene-specific crRNA were prepared as described in Cho et al. (2013). Briefly, the plasmid encoding His-tagged cas9 was expressed in *E. coli* and purified with Ni-NTA agarose resin (Qiagen), followed by dialysis. Synthesized complementary oligonucleotides for gene-specific crRNAs which were previously annealed *in vitro* and a cloned DNA corresponding tracrRNA were used as DNA templates for *in vitro* transcription by T7 RNA polymerase, followed by purification to remove unincorporated free NTPs. The RNA sequence of *sn*-specific crRNA and *e*-specific crRNA is 5'-CCA GCA CAA GUA CAU GAC CGG UUU UAG AGC UAU GCU GUU UUG-3', and 5'-UCU UCG AGG AGC AGC AGC UGG UUU UAG AGC UAU GCU GUU UUG-3', respectively, where the complementary sequences to the target DNA are underlined as in Fig. 1A and 3A.

Microinjection of *Drosophila* embryos

Gene-specific crRNA, tracrRNA and purified Cas9 protein were mixed with 2:2:1 molar ratio, based on our recent finding that the enhanced cleavage activity of RGEN is nearly saturated when the molar excess of sgRNA over Cas9 protein is over 2.5 fold higher (Kim et al., 2014b). Each component was mixed and incubated at 37℃ for 15 minutes to form the Cas9/tracrRNA/crRNA ribonucleoprotein complex before injection. Then, the complex diluted in 10mM Tris solution for the appropriate concentrations was injected into w^{1118} embryos with the concentrations based on crRNA as indicated in Table 1 using the standard *Drosophila* microinjection method.

Initially the amounts of crRNA injected together with Cas9 protein and tracrRNA were inferred from those of sgRNA used for mouse or zebrafish (1ng up to 100ng/ul per injected sgRNA, Sung et al., 2013), which turned out to be too low to be effective in *Drosophila* (for instance, 130ng injection of crRNA against *singed* produced no F1 mutants, Table 1). Thus, we increased the injection amounts to find the effective ranges for mutant generation up to the maximum concentration without solubility problem, which corresponds to 660ng/ul of crRNA (1320ng/ul of tracrRNA and 4000ng/ul of Cas9 protein, respectively, for 2:2:1 molar ratio) (Table 1).

Screening of mutant flies

In order to detect the mutant phenotypes of *ebony (e)* in F1 offspring, individual G0 founder flies were crossed with the 3rd chromosome balancers containing *e* mutations (*D/ e, TM3,Ser*) and looked for the *ebony* mutants with the TM3,Ser balancer phenotype in F1 generation. In case of *singed* (*sn)* gene, because *sn* is an X-linked gene, we crossed single G0 founder female flies injected with *sn*-specific crRNA–containing Cas9/RNA complex to w^{1118} male flies and scored *sn* mutants in F1 males. We screened approximately 60 F1 progenies per vial for the *e* mutation and 40 F1 males per vial for the *sn* mutation on average to identify flies carrying mutant phenotypes.

Analysis of induced mutations: the T7EI assay and direct sequencing

Mutations in *e* and *sn* loci induced by the injected Cas9/tracrRNA/crRNA complex were detected by the T7EI assay and/or direct sequencing. T7EI is an endonuclease enzyme that recognizes and cleaves bulged DNA structure due to the mismatched base pair (Kim et al., 2009). Genomic regions flanking the CRISPR/Cas9 target sites in *e* and *sn* loci were amplified with DNA extracted from G0 founders or F1 progeny harboring mutant phenotypes as templates using Direct PCR kit (Nanohelix). PCR primers used are as follows.

	***ebony* (*e*)**	
1st PCR	forward primer	reverse primer
	5'-TGGTGGATAACGTGGGAGTT-3'	5'-GCGCTCCATTAACACGAAGT-3'
2nd PCR	forward primer	reverse primer
	5'-CACAGCCACGATGAAGTCAC-3'	5'-GCATTAGCCTGCATTGCATA-3'

	***singed* (*sn*)**	
1st PCR	forward primer	reverse primer
	5'-CGTCCCACTCTCACATCCTT-3'	5'-CCCCGAATGACTGAATTGTT-3'
2nd PCR	forward primer	reverse primer
	5'-TTGCAGCACCATTAAGTTCG-3'	5'-CCAACTAACTAACATTTCTCCCATC-3'

The 1st PCR cycle was 95°C, 2min, 35cyles of (95°C, 30sec; 61°C, 30sec; 72°C, 30sec), 72°C, 5min. The 2nd PCR was performed using 1/100 dilution of the 1st PCR product as the template and the same PCR cycle used in the 1st PCR.

For the T7EI assay, purified PCR products were denatured/renatured in a temperature gradient of (95°C, 2min →∆ 2°C/sec to 85°C → ∆0.1°C/sec to 25°C → 16°C, forever) to allow the mismatched

DNA structure to be formed, followed by digestion with the T7EI enzyme (NEB # M0302) for 30min at 37°C and running on an 2~2.5% agarose gel for detection. For direct sequencing, sequences of PCR products themeshelves (in case of *sn*) or individual clones after PCR products were subcloned into *pCR®-BluntII-TOPO* vector (Invitrogen) (in case of *e*) were analyzed with conventional sequencing to find insertion/deletion mutations compared to the wild type sequences.
